# A Quality Improvement Project to Improve Syncope Care Through Structured Triage and Risk Stratification (STARS)

**DOI:** 10.7759/cureus.93043

**Published:** 2025-09-23

**Authors:** Aaron Lau, Minhaz Ahmed, Steve Parry

**Affiliations:** 1 Acute Medicine, Royal Victoria Infirmary, Newcastle upon Tyne, GBR

**Keywords:** cardiac risk factors and prevention, guideline, inpatient syncope workup, risk assessment tools, syncope workup

## Abstract

Background

Syncope is a common emergency presentation, with causes ranging from benign reflex syncope to high-risk cardiac conditions. The 2018 European Society of Cardiology (ESC) Guidelines recommend structured risk stratification to guide admission and telemetry, but real-world adherence remains inconsistent.

Objective

This Quality Improvement Project (QIP) evaluated the impact of a structured Syncope Risk Stratification Chart (SRSC) and targeted education on guideline adherence, clinical assessment, and resource use in our Emergency Department and Medical Admissions Unit (MAU).

Methods

We conducted a retrospective review of syncope admissions (n=100, April-June 2024), followed by interventions (SRSC dissemination, staff education, visual prompts). A prospective re-audit (n=29, May-June 2025) assessed documentation of lying/standing blood pressure (LSBP) and family history (FH), admission rates, and telemetry use.

Results

In Round 1, admission rates differed significantly by ESC risk group (81% low-risk, 96% medium-risk, 100% high-risk; χ²(2, N=100) = 9.84, p = 0.007). In Round 2, rates were 89%, 89%, and 100%, with no significant differences (χ²(2, N=29) = 1.31, p = 0.52). Overall admissions were unchanged (92% vs 93%, p = 1.00). Telemetry allocation remained inconsistent (R1: 17%, 71%, 71%; R2: 38%, 88%, 91%), with between-round differences not statistically significant. Documentation improved, with LSBP recording increasing from 59% to 72% (p = 0.27) and FH assessment from 15% to 41% (p = 0.005).

Conclusion

Implementation of the SRSC and education significantly improved family history documentation and modestly improved LSBP recording. However, admission and telemetry practices remained misaligned with ESC recommendations. Sustained system-level strategies, senior triage input, and integration of decision-support tools are needed to achieve consistent, guideline-based syncope care.

## Introduction

What is syncope?

According to the 2018 European Society of Cardiology (ESC) Guidelines, syncope is defined as a transient loss of consciousness (TLOC) due to “cerebral hypoperfusion, characterised by a rapid onset, short duration, and spontaneous recovery” [1]. Importantly, it occurs in the absence of focal neurological signs and typically does not require specific resuscitative measures [2].

Syncope is typically classified into two principal categories based on underlying aetiology [1]. Firstly, reflex syncope (RS), also known as neurally-mediated syncope, includes vasovagal syncope (VVS), situational syncope, and carotid sinus hypersensitivity (CSH). These are typically benign and result from transient dysfunction of autonomic control, leading to vasodilation and/or bradycardia.

Secondly, cardiac syncope (CS) arises from arrhythmias, structural cardiac abnormalities, or other significant cardiovascular conditions such as pulmonary embolism. CS is associated with the highest risk of adverse outcomes and therefore warrants urgent hospital admission and targeted treatment of the underlying pathology [1,3,4].

In addition to these, syncope due to orthostatic hypotension (OH) is recognised as a third distinct category. It is characterised by cerebral hypoperfusion secondary to an inadequate physiological response to upright posture, often due to autonomic failure, hypovolaemia, or pharmacologic effects [1].

Syncope statistics

Syncope is a frequent presentation to the ED [5], accounting for approximately 0.6-1.7% of attendances [1,6,7]. Admission rates following initial evaluation vary internationally, ranging from 12% to 86%, with an estimated admission rate of 49% in the United Kingdom [6,8]

The majority of syncope presentations are attributable to benign causes, with VVS and OH accounting for nearly two-thirds of presentations, while CS-although less prevalent-represents approximately 7-10% of presentations [2]. These findings are consistent with previous studies [4], which reported VVS in 53.3% of cases and CS in 5.4%. Notably, the prevalence of CS increases with age and is more commonly observed in older populations [2,6].

Despite advances in clinical assessment and diagnostic tools, a substantial proportion of patients remain without a definitive diagnosis following initial evaluation [6] and at the point of discharge [2], highlighting ongoing challenges in syncope management.

Financial statistics

Syncope imposes a substantial financial burden on healthcare systems. In the United States, inpatient evaluation for syncope is estimated to exceed $2.4 billion annually, with an average cost of $5,400 per hospitalisation [9]. Similarly, one study reported a mean cost per syncope admission of approximately $4,223 (£3,406), with a range of £2,000-£6,000 (conversion based on £1 = $1.24) [10]. When accounting for subsequent admissions, injuries, and outpatient visits, the total average cost per patient increased to $11,866 (£9,570). These numbers highlight the economic impact of syncope-related admissions and underscore the need for effective risk stratification and management strategies to reduce unnecessary hospitalisation.

Risk stratification

It is essential to differentiate syncope from other causes of TLOC, such as seizures, metabolic disturbances, or psychogenic episodes, as these conditions vary considerably in their underlying pathophysiology, risk profiles, and management strategies [11]. One key diagnostic challenge includes distinguishing convulsive syncope, which may occur in up to 70% of syncope patients, from primary seizure activity [2].

An accurate diagnosis not only ensures appropriate treatment and follow-up but also enables clinicians to clearly communicate the nature of the episode to patients. This is particularly important in cases such as psychogenic pseudosyncope, where specialist referral is often required [11,12]. While syncope may initially appear benign, clinicians must maintain a high index of suspicion for potentially serious, treatable cardiac causes [2].

Although RS and OH are generally considered more benign than CS, accurate triage and targeted preventative advice remain essential. OH, for instance, is associated with an increased risk of serious events within 30 days of ED presentation and is linked overall to a higher risk of cardiovascular morbidity and mortality [4,13]. Similarly, nearly 50% of patients with VVS experience at least one recurrence [14], further emphasizing the need for a structured risk assessment and patient education.

Current risk stratification scores

The initial assessment of syncope prioritises the identification of potentially life-threatening conditions [15] before proceeding to determine the underlying aetiology [11]. This process can be clinically challenging due to the broad differential diagnosis and the typically transient nature of symptoms. Additional challenges include variability in diagnostic strategies and yield among physicians, inconsistencies in clinical practice patterns, differences in resource availability, and varying degrees of adherence to established guidelines [1,11].

Several risk stratification tools have been developed to guide the management of syncope, such as the San Francisco Syncope Rule (SFSR) [16], the OESIL (Osservatorio Epidemiologico sulla Sincopenel Lazio) risk score [17], and the EGSYS (Evaluation of Guidelines in Syncope Study) score [18]. These tools were designed to assist clinicians in identifying patients at risk of serious outcomes and determining the need for admission. However, studies have shown that these scoring systems do not consistently outperform structured clinical judgement, even in predicting short-term adverse events [2,19].

The more recent Canadian Syncope Risk Score (CSRS) [20] has shown improved performance compared to earlier models and has been validated in external cohorts [21]. Nonetheless, its use is primarily recommended in cases of diagnostic uncertainty, underscoring the continued importance of comprehensive clinical evaluation and judgement in the assessment of syncope [22]. Despite these tools, structured clinical judgement remains the cornerstone of syncope assessment.

For this Quality Improvement Project (QIP), we adopted the ESC 2018 Guidelines for the Diagnosis and Management of Syncope as it is widely endorsed and utilised by cardiologists of the United Kingdom and represents the foundation of national syncope assessment pathways. These guidelines offer a comprehensive, evidence-based framework for evaluating patients with TLOC, emphasising structured clinical assessment, early identification of high-risk features, and appropriate risk stratification [1]. Importantly, one study demonstrated that the application of the ESC guidelines helped improve the diagnostic accuracy, from 65% under routine practice to 80% with a structured evaluation, without missing any life-threatening conditions [11]. This improvement was largely attributed to systematic history-taking, reinforcing its role as the cornerstone of syncope diagnosis

Problem description

The assessment and management of syncope within our Emergency Department (ED) and Medical Admissions Unit (MAU) lacked standardization and often deviated from established international guidelines, such as the ESC 2018 Guidelines. Awareness among medical and nursing staff regarding the prognostic importance of syncope and structured risk stratification was limited. Consequently, key risk factors were frequently omitted during assessment, leading to inconsistent documentation, variable triage decisions, and potentially avoidable hospital admissions. This contributed to inefficient use of the limited monitored beds.

Implementing a structured, algorithm-based approach is essential to support more consistent clinical decision-making, ensure appropriate risk-based triage, and reduce unnecessary admissions of low-risk patients. Equally, it enables prioritisation of telemetry for high-risk individuals who may require urgent cardiac monitoring or specialist input. This is especially important given the current pressures on hospital capacity, with adult general and acute wards often operating near full occupancy, many beds taken up by patients medically fit for discharge, and additional strain from seasonal illness surges. Optimising admission decisions and resource allocation is also vital for financial sustainability and for preventing further strain on cardiology services.

To our knowledge, no syncope-focused QIP addressing these specific challenges has been undertaken at our institution within the past few years, highlighting the need for local intervention.

Aims

The aim of our QIP was to enhance adherence to the ESC 2018 Syncope Guidelines for risk stratification and management of patients presenting with syncope in the ED and MAU. The objective was (by the end fo the project) to (i) improve the completeness of clinical assessment, with accurate documentation of lying/ standing blood pressure (LSBP) measurements and family history (FH) assessment, in line with ESC recommendations, (ii) ensure 100% of high-risk syncope patients are admitted with appropriate telemetry monitoring, and (iii) decrease unnecessary admissions among low-risk patients through the implementation of structured risk stratification.

## Materials and methods

**Figure 1:**
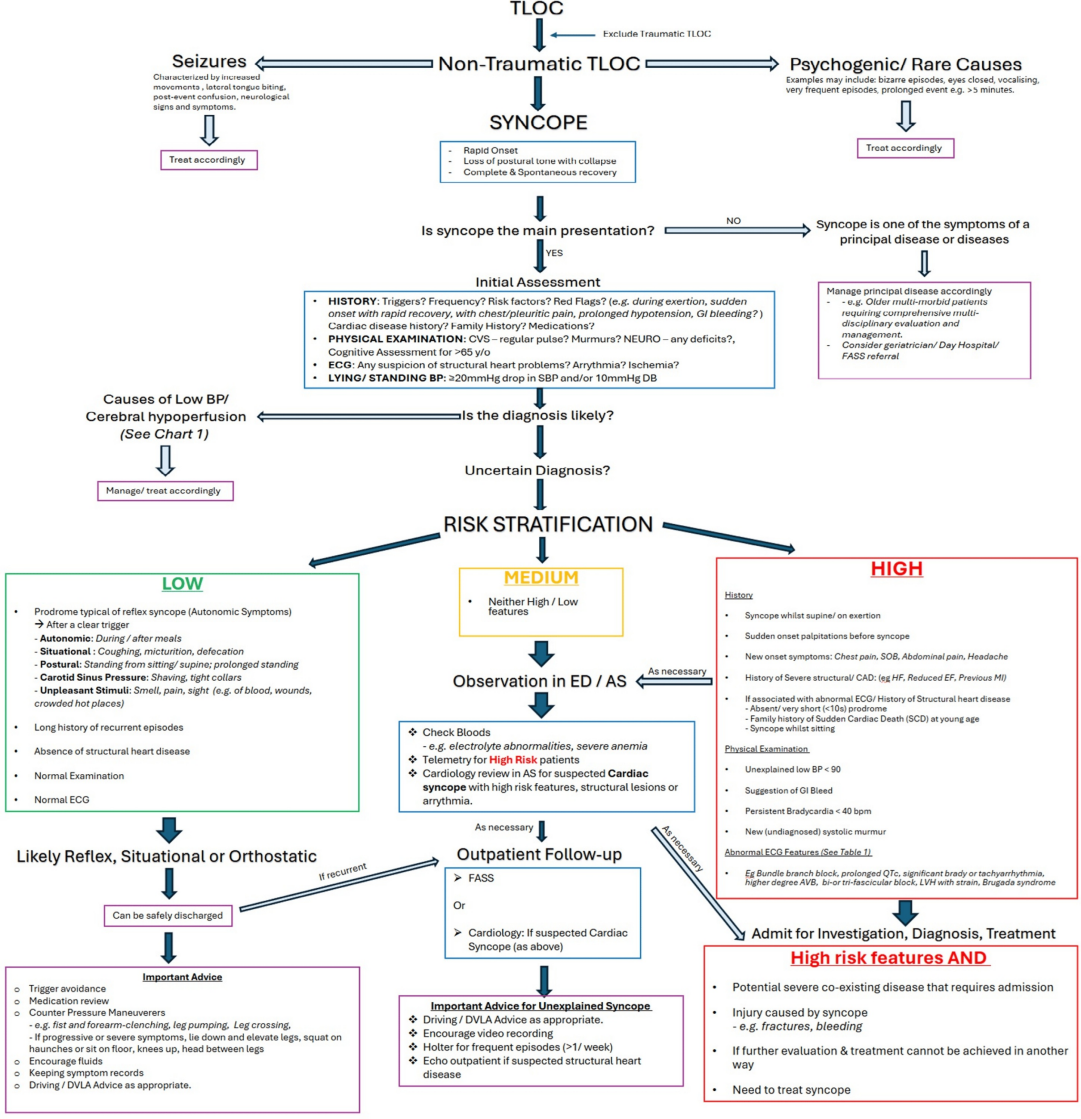
Syncope Risk Stratification Chart (SRSC) designed based on ESC 2018 Guidelines in collaboration with local cardiology and falls team. TLOC: transient loss of consciousness; CVS: cardiovascular; SOB: shortness of breath; CAD: coronary artery disease; AVB: atrioventricular block; LVH: left ventricular hypertrophy; DVLA: Driver and Vehicle Licensing Agency; HF: heart failure; EF: ejection fraction; FASS: Falls and Syncope Service; ESC: European Society of Cardiology

This QIP was conducted at Royal Victoria Infirmary, Newcastle upon Tyne, United Kingdom. Ethical approval for this QIP was obtained from Newcastle upon Tyne Hospitals NHS Foundation Trust's information governance department (reference number: 16803). Members of the project team were involved in the development and implementation of the Syncope Risk Stratification Chart (SRSC) (Figure 1) as part of a QIP aimed at improving adherence to the ESC 2018 Syncope Guidelines.

Study design and sampling

This project was conducted as a two-cycle quality improvement study with retrospective (R1) and prospective (R2) audit components. A formal sample size calculation was not undertaken, as is standard for quality improvement work; instead, we reviewed all consecutive patients meeting eligibility criteria during predefined audit windows.

Inclusion criteria were: all patients presenting to the ED and MAU with syncope between April-June 2024 (R1) and May-June 2025 (R2). Exclusion criteria were: patients in whom transient loss of consciousness was attributable to other causes (e.g., seizure, hypoglycaemia, intoxication), those transferred from other hospitals, and cases with incomplete records preventing adequate risk stratification.

This approach ensured consecutive sampling, minimised selection bias, and reflected real-world clinical practice within the institution.

Audit rounds

In the first audit cycle (Round 1, R1), a retrospective review was conducted on 100 consecutive patients presenting with syncope to the ED and MAU between April and June 2024. Electronic patient records were examined to extract data on clinical history, physical assessment, triage categorisation, investigations undertaken, and subsequent management decisions. The second cycle (Round 2, R2), following implementation of interventions, consisted of a prospective review of syncope admissions between May and June 2025.

For both cycles, patients were stratified into low-risk, intermediate-risk, or high-risk categories according to the criteria outlined in the 2018 ESC Syncope Guidelines. Patterns of investigation, utilisation of telemetry monitoring, admission rates, and final diagnoses were then analysed in relation to these risk classifications.

Interventions

Following the first audit cycle (R1), data were compiled and categorised to enable structured analysis. Subsequently, several targeted interventions were implemented: (i) Electronic distribution of the SRSC (Figure 1) to medical staff working on the MAU and ED, (ii) Educational sessions for MAU and ED staff, highlighting audit findings and introducing the SRSC, (iii) Display of printed SRSC posters in the doctors’ handover room and clinical workstations, and (iv) Presentation of findings and discussion regarding SRSC implementation at a local departmental meeting involving senior medical and ED staff.

Intervention strategies were selected based on the operational structure of the MAU and ED. Email dissemination was employed to maximise coverage, recognising that junior doctors frequently rotate through these departments. This ensured that all relevant clinicians, including both incoming and outgoing staff, were aware of the SRSC and associated guidance. Morning teaching sessions provided timely education to the working team of the day, reinforcing awareness of the ESC 2018 Syncope Guidelines and the implementation of the SRSC. This method aligned with evidence showing that clinical performance feedback and benchmark comparisons enhance healthcare professionals’ intentions to improve practice [23].

To support further adherence, poster handouts were prominently displayed as visual prompts. These prompts and cues can improve compliance with clinical protocols and facilitate behavioural change in acute care settings, especially when they are visible, timely, and placed near decision points.

Furthermore, senior medical staff in the ED and MAU were prioritised for direct communication, given their extended presence on the wards and their key role in supervising and advising junior staff. Their familiarity with departmental workflow makes them well-positioned to provide individualised, opportunistic teaching and reinforce consistent practice during clinical supervision.

Statistical analysis

Categorical variables (e.g., admission rates, telemetry use, documentation of LSBP and FH) were compared using chi-square (χ²) tests of independence, with Fisher’s exact test applied when expected counts were <5. Continuous variables (e.g., age) were compared between audit cycles using independent-samples t-tests (Welch’s correction applied if variances were unequal). Results are presented with test statistics, degrees of freedom, 95% confidence intervals (CIs), and p-values. Statistical significance was defined as p<0.05.

## Results

Patient demographics

In R1, 59% of patients were male and 41% female. In R2, 72% were male and 28% female. The difference in sex distribution between rounds was not statistically significant (χ²(1, N=129) = 1.19, p = 0.27). The mean age was 72.6 years (SD 16.2) in R1 and 70.8 years (SD 16.7) in R2. The age distribution did not differ significantly between rounds (t(44.4) = 0.51, p = 0.61; mean difference 1.8 years, 95% CI -5.3 to 8.8). Tables 1, 2 show the sex and age statistics for RI and R2, respectively.

**Table 1:** Patient demographics and age statistics for Round 1 (N=100) Sex distribution compared using χ² test (df=1). Statistical significance was defined as p<0.05.

Characteristics	Value
Sex, n (%)	
Male	59 (59%)
Female	41 (41%)
Age (Years)	
Mean±SD	72.59±16.17
Median (range)	76 (23 – 98)
Variance	261.58

**Table 2:** Patient demographics and age statistics for Round 2 (N=29)

Characteristics	Value
Sex, n (%)	
Male	21 (72%)
Female	8 (28%)
Age (Years)	
Mean ± SD	70.8 ± 16.7
Median (range)	72 (22 – 99)
Variance	278.9

Admission rates

In R1, admission rates differed significantly across ESC risk groups: 81% of low-risk, 96% of medium-risk, and 100% of high-risk patients were admitted (χ²(2, N=100) = 9.84, p = 0.007). In contrast, in R2, admission rates were 89%, 89%, and 100% respectively, with no significant differences between groups (χ²(2, N=29) = 1.31, p = 0.52). Comparing between rounds, overall admission rates did not change (92% in R1 vs 93% in R2; χ²(1, N=129) = 0.00, p = 1.00). Admission rates for individual risk groups also did not differ significantly (low-risk: χ²(1) = 0.004, p = 0.95; medium-risk: χ²(1) = 0.00, p = 1.00; high-risk: 100% in both rounds).

Telemetry allocation

In R1, telemetry allocation differed significantly across risk categories: 17% of low-risk, 71% of medium-risk, and 71% of high-risk admitted patients received telemetry (χ²(2, n=92) = 23.95, p < 0.001). In R2, telemetry use was 38% in low-risk, 88% in medium-risk, and 91% in high-risk admitted patients, with significant variation across groups (χ²(2, n=27) = 7.95, p = 0.019). Between rounds, telemetry use increased but did not reach statistical significance within individual risk strata (low-risk: χ²(1) = 0.63, p = 0.43; medium-risk: χ²(1) = 0.22, p = 0.64; high-risk: χ²(1) = 0.90, p = 0.34). Tables 3, 4 show the admissions and telemetry use by ESC risk group in R1 and R2, respectively.

**Table 3:** Admissions and telemetry use by ESC risk group in Round 1

Risk group	Number of patients, n (%)	Number of patients admitted, n (%)	Number of admitted patients on telemetry, n (%)
Low-risk	37 (37%)	30 (81%)	5 (17%)
Medium-risk	25 (25%)	24 (96%)	17 (71%)
High-risk	38 (38%)	38 (100%)	27 (71%)

**Table 4:** Admissions and telemetry use by ESC risk group in Round 2

Risk group	Number of patients, n (%)	Number of patients admitted, n (%)	Number of admitted patients on telemetry, n (%)
Low-risk	9 (31%)	8 (89%)	3 (38%)
Medium-risk	9 (31%)	8 (89%)	7 (88%)
High-risk	11 (38%)	11 (100%)	10 (91%)

LSBP and FH documentation

Documentation of LSBP improved from 59% in R1 to 72% in R2, though this was not statistically significant (χ²(1, N=129) = 1.19, p = 0.27). FH assessment increased significantly from 15% in Round 1 to 41% in Round 2 (χ²(1, N=129) = 7.93, p = 0.005). Table 5 shows the LSBP and family history before R1 and after R2 interventions.

**Table 5:** Lying/standing blood pressure (LSBP) and family history before Round 1 and after Round 2 interventions

	Before intervention - Round 1, n (%)	After intervention - Round 2, n (%)
LSBP documented	59 (59%)	21 (72%)
Family history assessed	15 (15%)	12 (41%)

## Discussion

The majority of patients in our sample were elderly, with a mean age above 70 years across both cycles. This is consistent with wider literature, which consistently reports a higher prevalence of syncope among older adults [24,25]. This demographic detail is clinically significant, as elderly patients with syncope face increased risks of morbidity, mortality, and fall-related injuries, and are prone to misclassification as mechanical falls [26,27]. Conversely, in younger patients, particularly those with orthostatic hypotension, unnecessary admissions may be avoided, as simple, low-cost interventions like counterpressure manoeuvres can prevent recurrence [28], reinforcing the need for tailored, risk-based triage [1].

Despite targeted interventions, admission practices remained unchanged. In R1, 81% of low-risk, 96% of medium-risk, and 100% of high-risk patients were admitted, while in R2, the corresponding figures were 89%, 89%, and 100%. Overall admission rates did not differ between rounds (92% vs 93%, p = 1.00). Notably, admission rates in the low-risk group remained high (81% vs 89%, p = 0.95), highlighting a persistent disconnect between practice and guideline recommendations, which advise safe discharge with safety-netting and lifestyle advice for low-risk patients (Figure 1). This suggests that real-world triage continues to be influenced by factors beyond clinical risk, such as medico-legal concerns, clinician uncertainty, and systemic pressures, including bed management policies.

Telemetry allocation also remained inconsistent. In R1, only 17% of low-risk, but 71% of medium- and high-risk admitted patients received telemetry. In R2, use increased across all groups (38%, 88%, and 91% respectively), with significant variation between categories. However, when comparing between rounds, improvements within each risk group were not statistically significant. This highlights the ongoing challenge of optimising limited monitoring resources, with persistent overuse in low-risk cases and underuse in some high-risk patients. These patterns likely reflect both the constraint of limited telemetry bed capacity and confidence gaps among junior clinicians, who may request telemetry for low-risk patients as a precaution in the absence of senior input.

The small sample size in R2 likely limited the power to demonstrate statistically significant changes in admission and telemetry practices, even though upward trends were observed. The increase in telemetry allocation and consistently high admission rates may also reflect greater clinician awareness following targeted interventions. This is supported by the concurrent improvement in documentation and history taking. A larger sample and longer re-audit cycle would be required to determine whether these early trends translate into sustained, measurable changes in practice.

In contrast, documentation practices showed meaningful improvement. FH assessment increased significantly from 15% in R1 to 41% in R2 (p = 0.005), while LSBP documentation improved from 59% to 72% (p = 0.27). These findings demonstrate that simple interventions-such as education, visual prompts, and structured tools-can enhance adherence to guideline-recommended assessments.

Overall, our results indicate that while structured interventions improved documentation, they were insufficient to change admission and monitoring practices. Achieving alignment with ESC recommendations is likely to require sustained system-level strategies. Real-time senior input during triage, decision-support integration, better structured outpatient pathways [2], dedicated syncope observation pathways [15,29] may be necessary to reduce unnecessary admissions, optimise telemetry use, and improve patient outcomes.

In addition to improving documentation, our targeted interventions proved practical and well-received. To support sustainability, these measures have now been incorporated into departmental induction materials for incoming junior doctors in the MAU and ED. Embedding the SRSC and associated education into routine induction offers a structured way to maintain awareness, promote consistency in practice, and gradually align local care pathways with guideline-based recommendations. 

Limitations and future recommendations

This QIP has several limitations. First, important patient-level factors such as frailty, functional status, and baseline mobility were not assessed, despite their likely influence on admission decisions. Second, operational variables, including time of presentation (day vs night), waiting times, and access to senior review, were not captured, even though they may have affected management pathways. Third, some admissions occurred for non-clinical reasons, such as delays in allied health or social care assessments, which are common in older populations and may have inflated admission rates. We also did not capture the underlying reasons for admitting low-risk patients (e.g., social factors, junior versus senior decision-making, or system pressures), which may have provided further insight into strategies for reducing unnecessary admissions. The relatively small sample size in the second audit cycle (n=29) limits statistical power, particularly for subgroup comparisons of admission and telemetry practices. As a single-centre project, the findings may also not be generalisable to other institutions. However, despite these limitations, the project highlights persistent gaps between guideline recommendations and clinical practice, while showing that targeted, low-cost interventions can improve documentation and awareness. Future work should include broader patient- and system-level variables and evaluate sustained, system-wide interventions in larger cohorts.

## Conclusions

This QIP demonstrated that simple, structured interventions, such as the SRSC and targeted education, can significantly improve documentation of key clinical assessments, particularly FH. However, admission and telemetry practices remained inconsistent with ESC guideline recommendations, highlighting a persistent gap between evidence and practice.

Closing this gap requires system-level strategies beyond education alone, including embedding decision-support tools into electronic records, ensuring real-time senior involvement in triage, and developing syncope observation pathways, which are likely to be essential. Sustained multidisciplinary engagement and digital integration of risk tools will be key to reducing unnecessary admissions, optimising use of limited monitoring resources, and improving patient outcomes in syncope care.
